# Citrate confers less filter-induced complement activation and neutrophil degranulation than heparin when used for anticoagulation during continuous venovenous haemofiltration in critically ill patients

**DOI:** 10.1186/1471-2369-15-19

**Published:** 2014-01-17

**Authors:** Louise Schilder, S Azam Nurmohamed, Pieter M ter Wee, Nanne J Paauw, Armand RJ Girbes, Albertus Beishuizen, Robert HJ Beelen, AB Johan Groeneveld

**Affiliations:** 1 Department of Nephrology, VU University Medical Center, Amsterdam, The Netherlands; 2 Department of Molecular Cell Biology and Immunology, VU University Medical Center, Amsterdam, The Netherlands; 3 Department of Intensive Care, VU University Medical Center, Amsterdam, The Netherlands; 4 Department of Intensive Care, Erasmus Medical Center, Rotterdam, The Netherlands

**Keywords:** Acute kidney injury, Biocompatibility, Citrate, Complement activation, Critically ill patients, Elastase, Heparin, MPO, Neutrophils, Renal replacement therapy

## Abstract

**Background:**

During continuous venovenous haemofiltration (CVVH), regional anticoagulation with citrate may be superior to heparin in terms of biocompatibility, since heparin as opposed to citrate may activate complement (reflected by circulating C5a) and induce neutrophil degranulation in the filter and myeloperoxidase (MPO) release from endothelium.

**Methods:**

No anticoagulation (n = 13), unfractionated heparin (n = 8) and trisodium citrate (n = 17) regimens during CVVH were compared. Blood samples were collected pre- and postfilter; C5a, elastase and MPO were determined by ELISA. Additionally, C5a was also measured in the ultrafiltrate.

**Results:**

In the heparin group, there was C5a production across the filter which most decreased over time as compared to other groups (P = 0.007). There was also net production of elastase and MPO across the filter during heparin anticoagulation (P = 0.049 or lower), while production was minimal and absent in the no anticoagulation and citrate group, respectively. During heparin anticoagulation, plasma concentrations of MPO at the inlet increased in the first 10 minutes of CVVH (P = 0.024).

**Conclusion:**

Citrate confers less filter-induced, potentially harmful complement activation and neutrophil degranulation and less endothelial activation than heparin when used for anticoagulation during continuous venovenous haemofiltration in critically ill patients.

## Background

Acute kidney injury (AKI) is common in critically ill patients and carries a high mortality rate [1]. To temporarily replace renal function, continuous venovenous haemofiltration (CVVH) is often used. Anticoagulation is required to prevent clotting of the extracorporeal system. Frequently used modes of anticoagulation include systemic heparin and regional citrate administration. Heparin is contraindicated in patients with a high bleeding tendency or heparin-induced thrombocytopenia and regional anticoagulation by citrate, in which only the extracorporeal circuit is anticoagulated by chelation of calcium, can then be used safely and effectively [2,3]. Potential benefits of the latter may include prolonged filter survival times and improved biocompatibility of the filter and this may help to explain the lower mortality rates of citrate-CVVH found by others [3,4].

Bioincompatibility is best described by an inflammatory response to various components of CVVH [5-7], including complement activation and C5a release followed by polymorphonuclear cell degranulation with release of intracellular products such as elastase, complexed in the circulation to alpha1-antitrypsin, and myeloperoxidase (MPO). The type of anticoagulation could contribute to the magnitude of the inflammatory response. As demonstrated in intermittent haemodialysis and in blood in vitro, calcium-chelating citrate anticoagulation may lower polymorphonuclear cell degranulation as observed with heparin; this may be partly independent of complement activation since cations, such as calcium, may be pivotal in cell activation and degranulation [8-15]. Nevertheless, the use of citrate may also prevent activation of complement by blood-membrane contact and endothelial release of MPO, as seen during heparin anticoagulation [16]. The single prior, albeit small, study suggesting prevention of neutrophil degranulation with citrate-CVVH as compared to heparin lacked a control group (no anticoagulation) and measurements of complement activation products [15].

We therefore hypothesized that regional anticoagulation with citrate causes less complement activation, polymorphonuclear cell degranulation and endothelial MPO release as compared to heparin, during CVVH in critically ill patients with AKI. We studied these variables for the first filter applied in critically ill patients with AKI in need of renal replacement therapy and who were either not anticoagulated or anticoagulated with citrate or heparin.

## Methods

The patient population and methods for this study are reported elsewhere [3,7]. In brief, the first study started one year before the availability of a custom-made citrate-based replacement fluid. From March 2004 to September 2005, patients admitted to the ICU who developed AKI necessitating CVVH but did not receive heparin due to a high bleeding tendency, either received anticoagulant-free CVVH (n = 13) or, after becoming available in 2005, regional citrate anticoagulation (n = 10) and were prospectively followed. High risk for bleeding was arbitrarily defined as a platelet count of less than 40 x10^9^/L, an activated partial thromboplastin time (aPTT) of more than 60 seconds, a prothrombin time of more than 2.0 international normalized ratio, a recent major bleeding, or active bleeding. Because all patients were treated according to local standards, the need for informed consent was waived for this study. Patients were also recruited from a second study which is a randomized controlled trial initiated in 2006 (the so called ‘Citrate anticoagulation versus systemic heparinization’, CASH trial, clinicaltrials.gov number NCT00209378, manuscript in preparation). Patients admitted to the ICU who developed AKI necessitating CVVH without a high bleeding risk, were randomized between unfractionated heparin as anticoagulant (n = 8) or citrate (n = 7). Informed consent was obtained from all study participants or their next-of-kin. Study protocols were approved by the local medical ethical committee and performed in accordance with the Declaration of Helsinki.

### Treatment protocol

The indication for CVVH was based on standard clinical indications that include AKI accompanied by haemodynamic instability, ongoing hypercatabolism, diuretic-resistant fluid overload, respiratory distress, multiorgan failure, or some combinations of these factors. CVVH was performed using a haemofiltration machine (DIAPACT, B. Braun Medical, Melsungen, Germany). Vascular access was obtained by the insertion of an 11 F double-lumen catheter (GAMCATH, Gambro, Hechingen, Germany) into the femoral, subclavian, or internal jungular vein. A 1.9 m^2^ highly permeable cellulose triacetate filter (NIPRO UF-205, Nissho Corp, Osaka, Japan, cutoff approximately 40 kDa) was used in all treatments. For lactate- or bicarbonate-based CVVH, commercially prepared buffer solutions were used (BH504 [lactate-based] or HF32bic [bicarbonate-based] in n = 6 Group 1 and n = 5 in Group 2, Dirinco, Rosmalen, the Netherlands); the lactate solution contained 140 mmol/L sodium, 1.5 mmol/L potassium, 1.5 mmol/L calcium, 103 mmol/L chloride, 11.1 mmol/L glucose, and 42 mmol/L lactate; the bicarbonate solution contained 140 mmol/L sodium, 2.0 mmol/L potassium, 0.5 mmol/L magnesium, 1.75 mmol/L calcium, 111.5 mmol/L chloride, 1.0 mmol/L glucose, 32.0 mmol/Lbicarbonate and 3.0 mmol/L lactate. Patients with high serum lactate levels (>5 mmol/L) were routinely treated with bicarbonate-buffered rather than lactate buffered CVVH. For the use of citrate, a replacement solution was custom-made by Dirinco (Rosmalen, the Netherlands), according to the protocol described before [3]. The solution contained 139.9 mmol/L sodium, 3.0 mmol/L potassium, 0.5 mmol/L magnesium, no calcium, 104 mmol/L chloride, 5 mmol/L glucose and 13.3 mmol/L citrate. Blood flow rate was set at 180 mL/min in all groups. Replacement fluid was administered at a standard rate of 2000 mL/h in the patients receiving no anticoagulation or heparin with bicarbonate or lactate-containing replacement fluids. Patients on heparin were administered a heparin bolus of 5000 IU followed by a body weight based continuous infusion in order to reach an activated partial thromboplastin time (aPTT) between 55–65 seconds. Replacement fluid rates in the citrate group were set at 2400 mL/h. Replacement fluids were infused in predilutional mode. Patients receiving citrate-based therapy had a separate intravenous infusion with calcium glubionate (Calcium Sandoz, containing 0.225 mmol/mL calcium, Novartis Consumer Health, Breda, The Netherlands). Calcium administration was adapted to concentrations of ionized calcium in the patient by a specially designed algorithm, as described before [3]. The target ionized calcium concentration in the circuit was 0.3 mmol/L, but not routinely monitored since this is almost uniformly achieved with the indicated settings [3].

### Study protocol

At inclusion, demographic variables were recorded such as age, gender and reason of intensive care unit (ICU) admission. Assessment of disease severity on ICU admission was done according to the simplified acute physiology score II (SAPS II) and the sequential organ failure assessment score (SOFA). We collected systemic inflammatory response syndrome (SIRS) criteria: body temperature >38°C; a heart rate of >90 beats/min; a respiratory rate of >20 breaths/min or mechanical ventilation; and white blood cell counts of <4.0 *10^9^/L or >12.0 *10^9^/L. When SIRS (2 or more criteria) and an infection were present (either clinically suspected or microbiologically confirmed), patients were classified as having sepsis. Venous blood samples were collected from the haemofiltration catheter from each patient before the initiation of CVVH and administration of heparin. Heparin was given immediately after filter connection. Thereafter, blood samples were collected after 10, 60, 180 and 720 min from the pre- and postfilter pole. Prefilter blood was drawn before addition of replacement fluids. Leukocytes, platelets, aPTT and serum creatinine concentrations were measured before initiation of CVVH and after 720 minutes. In all patients, a zero fluid balance was achieved during the time points at which blood samples were drawn. Samples were collected in standard Vacutainer tubes (Becton, Dickinson and Company, Erembodegem, Belgium) with ethylenediaminetetraacetic acid (EDTA), benzamidine and soybean trypsin inhibitor added. For C5a, ultrafiltrate samples were collected from the appropriate ports. Samples were centrifuged at 1,300 g for 10 minutes at 4°C and stored at -80°C until assayed.

### Measurements and calculations

C5a concentrations were measured by enzyme-linked immunosorbent assays (ELISAs). We measured concentrations of the relatively small molecule C5a (11 kDa) in the ultrafiltrate to assess whether C5a is removed from the circulation during CVVH, and whether the anticoagulation applied influences this. Commercially available antibody duosets were used (R&D Systems, UK, Human Complement Component C5a Duoset, DY2037). Plasma samples and ultrafiltrate were measured in separate assays with C5a standards prepared in a 0.5% bovine albumin serum phosphate buffered saline, 0.05% Tween solution or fresh ultrafiltrate as appropriate. The formulas used to assess removal of C5a from the circulation are described in Formulas used to evaluate fluxes. Elastase and MPO were measured by commercially available ELISAs employing a monoclonal capture antibody to polyclonal detecting antibody with a peroxidase-detecting system. For elastase (Hycult, The Netherlands, Human Elastase ELISA kit, HK313) as well as for MPO (Hycult, The Netherlands, Human MPO ELISA kit, HK324), the lower detection limit was 0.4 ng/mL. All measurements were done according to the protocols provided by the manufacture. Each sample was run in duplicate and the mean concentration was calculated. Formulas used to evaluate fluxes are described below. Concentrations of MPO and elastase in the ultrafiltrate were undetectable.

### Formulas used to evaluate fluxes

$$\begin{array}{l} Q_{i}=Q_{b}\times \left(1-\mathrm{Ht}\right),Q_{o}=Q_{i}\\ \mathrm{Mi}=Q_{i}\times C_{i}\\ M_{o}=Q_{o}\times C_{o}\\ M_{\mathrm{uf}}=Q_{\mathrm{uf}}\times C_{\mathrm{uf}}\\ M_{\mathrm{tp}}=\left(M_{o}+M_{\mathrm{uf}}\right)-M_{i}\\ C=C_{i}\times Q_{i}/\left(Q_{i}+\mathrm{RF}\right)\\ \mathrm{SC}=2\times C_{\mathrm{uf}}/\left(C+C_{o}\right) \end{array}$$

For elastase and myeloperoxidase:

$$M_{\mathrm{tp}}=M_{o}-M_{i}$$

Abbreviations: C_i_ Concentration in inlet plasma before addition of replacement fluid, C_o_ Concentration in outlet plasma, C_uf_ Concentration in ultrafiltrate, Q_b_ Inlet blood flow rate, Q_i_ Inlet plasma flow rate, Q_o_ Outlet plasma flow rate, Q_uf_ Ultrafiltration flow rate, M_i_ Mass inlet rate, M_o_ Mass outlet rate, M_uf_ Mass ultrafiltration rate, M_tp_ Mass production rate, C Concentration in inlet plasma after addition of replacement fluid, RF Replacement fluid flow rate, SC Sieving coefficient.

### Statistical analysis

Due to the non-Gaussian distributions, data are presented as median and range. Since there were no baseline differences between the two separate citrate groups (citrate vs. no-anticoagulation and citrate vs. heparin, Table 1) and there were no important differences between the groups in courses of concentrations of C5a, elastase and MPO over time (See Additional file 1), the citrate data were pooled. Group differences were evaluated using the Kruskal-Wallis or Χ^2^ tests. Groups were compared for data at individual time points using the Mann–Whitney U test. To evaluate differences according to anticoagulation regimens in time we used generalized estimating equations (GEE) taking repeated measurements in the same patient into account. The focus of GEE is on estimating differences between anticoagulation groups and time points, and their first order interaction, i.e. differences between anticoagulation groups over time, and associated P values are reported. When appropriate, data were log-transformed to achieve normal distributions (Kolmogorov-Smirnov test P > 0.05). The values for the total mass production rate were ranked, since some of the values were negative and could not be log-transformed. Spearman’s correlation coefficient was used to express relations. A P <0.05 was considered statistically significant. Exact P values are given unless <0.001.

**Table 1:** Characteristics of patients anticoagulated with citrate from two different studies

	**Observational study (n = 10)**	**Randomized study (n = 8)**	**P**
Age, years	66 (32–79)	56 (42–74)	0.54
Sex, male	5 (50)	6 (86)	0.30
Weight, kg	75 (60–100)	80 (60–110)	0.48
**Reason of admission:**			0.10
Respiratory failure	5 (50)	0	
Circulatory failure	1 (10)	1 (14)	
Trauma	1 (10)	0	
Post-resuscitation	0	2 (29)	
Surgery	3 (30)	4 (57)	
Sepsis	6 (60)	1 (14)	0.13
SAPS II	50 (32–60)	62 (38–86)	0.06
SOFA	13 (8–18)	14 (9–15)	0.81
Mechanical ventilation	9 (90)	7 (100)	1.00
Vasopressor dependent	8 (80)	5 (71)	1.00
Mortality in ICU	6 (60)	6 (86)	0.34

Median (range) or number (percentage) where appropriate. SAPS II: Simplified Acute Physiology Score II; SOFA: Sequential Organ Failure Assessment score; ICU = Intensive care unit.

## Results

Patient characteristics are presented in Table 2. Thirteen patients were treated by anticoagulant-free CVVH, 8 patients by heparin and 17 patients by citrate-CVVH. At baseline, all patients met the criteria for SIRS and 17 patients met the criteria for sepsis. The no anticoagulation group had a higher SAPS II score than the other groups. Values of leukocytes, platelets, creatinine and aPTT are presented in Table 3. The creatinine concentration at baseline was lower in the no anticoagulation group than in the other groups.

**Table 2:** Patient characteristics in the anticoagulation groups

	**No anticoagulation n = 13**	**Heparin n = 8**	**Citrate n = 17**	**P**
Age (years)	70 (34–84)	57 (23–81)	61 (32–79)	0.41
Sex (male)	7 (54)	6 (75)	11 (65)	0.61
Weight (kg)	70 (50–100)	73.5 (55–135)	75 (60–110)	0.50
**Reason of admission:**				0.27
Respiratory failure	2 (15)	5 (63)	5 (29)	
Circulatory failure	3 (23)	1 (13)	2 (12)	
Trauma	2 (15)	0	1 (6)	
Post-resuscitation	2 (15)	0	2 (12)	
Surgery	4 (31)	1 (13)	7 (41)	
AKI	0	1 (13)	0	
Sepsis	5 (39)	5 (63)	7 (41)	
SAPS II	75 (43–112)	47 (37–77)	52 (32–86)	<0.001
SOFA	14 (7–21)	11 (8–15)	13 (8–18)	0.02
Mechanical ventilation	12 (93)	5 (63)	7 (41)	0.74
Norepinephrine (μg/kg/min)	0.72 (0–2.8)	0.15 (0–1.2)	0.12 (0–1.1)	0.20
ICU stay (days)	5 (2–82)	19.5 (4–48)	20 (3–81)	0.11
Mortality in ICU	9 (69)	1 (13)	5 (29)	0.02

Median (range) or number (percentage) where appropriate. SAPS II: Simplified Acute Physiology Score II; SOFA: Sequential Organ Failure Assessment score; ICU = Intensive care unit.

**Table 3:** Laboratory data

	**No anticoagulation n = 13**	**Heparin n = 8**	**Citrate n = 17**	**P**
Leukocytes (×10^9^/L)				
0 h	7.8 (1–17.2)	11.6 (6.5-19.7)	11.4 (1.3-25.7)	0.33
12 h	8.1 (1.2-24.8)	12.4 (5.2-24.2)	11.6 (0.8-116)	0.39
Platelets (×10^9^/L)				
0 h	65 (22–777)	167 (44–332)	117 (35–332)	0.03
12 h	72 (8–131)	140 (45–203)	100 (18–335)	0.04
aPTT(sec)				
0 h	48 (39–72)	40 (36–81)	48 (34–94)	0.56
12 h	46 (41–118)	62 (40–125)	46 (33–68)	0.03
Creatinine (μmol/L)				
0 h	249 (100–410)	420 (156–626)	311 (47–626)	0.02
24 h	206 (140–250)	284 (125–463)	236 (46–441)	0.04

Median (range). aPTT = activated partial prothrombin time.

### C5a

**Figure 1:**
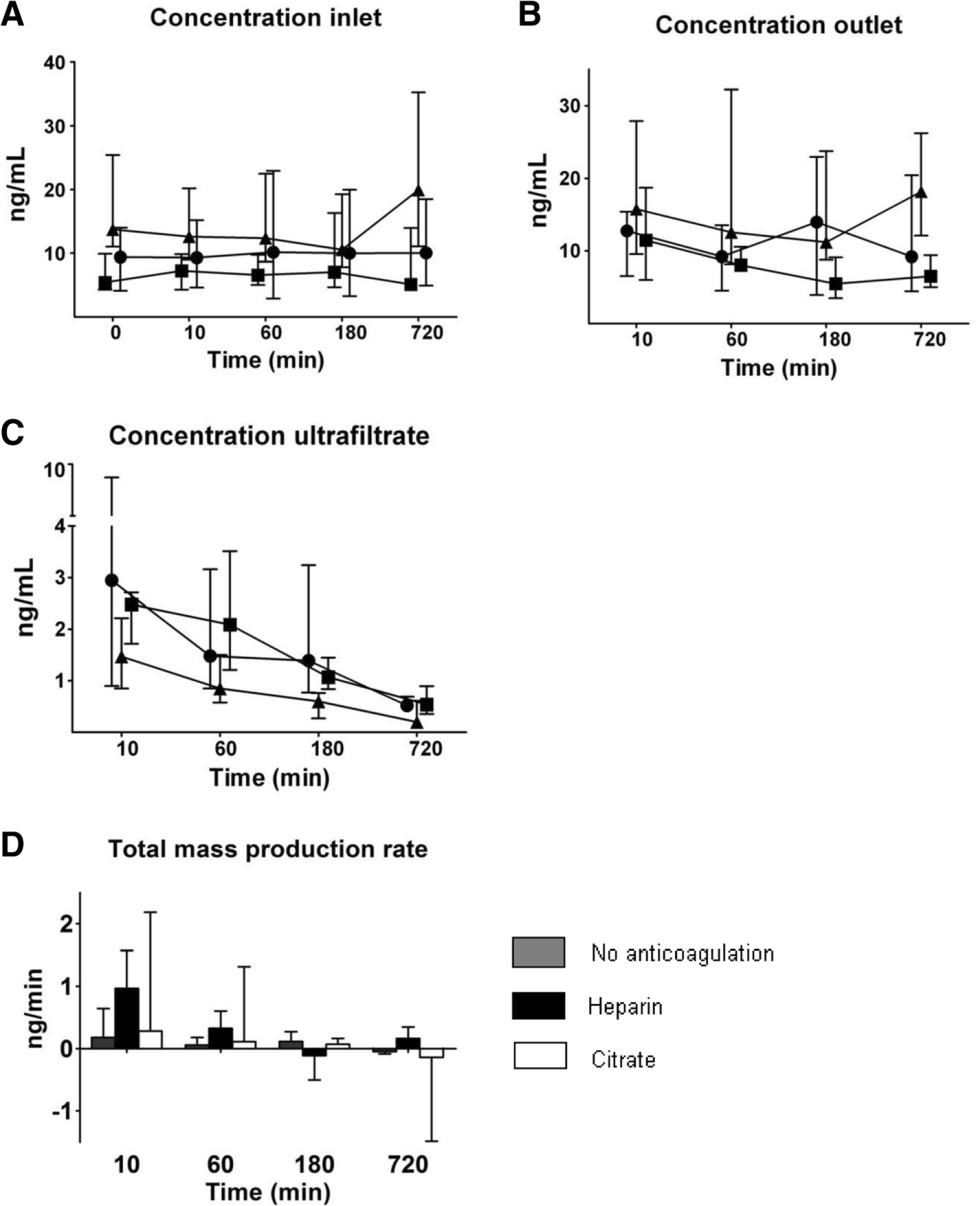
**Concentration of complement product C5a measured at inlet (C**_**i**_**), outlet (C**_**o**_**), in the ultrafiltrate (C**_**uf**_**) and total mass production rate (M**_**tp**_**) over time (median and interquartile range).** Results of generalized estimating equations (symbols: ● no anticoagulation ■ heparin ▲ citrate) for C5a in **A.** C_i_: highest in the citrate group (P = 0.016). **B.** C_o_: highest in the citrate group (P = 0.026), decreases over time (P = 0.012) but least in the citrate group (P = 0.008). **C.** C_uf_: lowest in the citrate group (P < 0.001), decreases over time (P < 0.001). **D.** M_tp_: decreases over time (P < 0.001), most in the heparin group (P = 0.007 for interaction).

Prior to initiation of CVVH, C5a was detectable in all patients at the inlet. Before initiation of CVVH, the concentration in inlet plasma was already higher in the citrate than in the other groups (P = 0.009) [Figure 1]. Also, the outlet concentration was higher in the citrate group and decreased in time but less so in the citrate group than the other groups. C5a was detectable in 92% of the total measurements in the ultrafiltrate; the concentration was lowest (albeit different, Additional file 1) in the citrate groups and decreased in time in all groups. The sieving coefficient was low and lower in the citrate than the other groups (no anticoagulation 0.49 (0.05-9.45), heparin 0.35 (0.12-11.2), citrate 0.10 (0.02-0.62), P < 0.001) and decreased in time in all groups (P < 0.001). Therefore, the clearance of C5a, calculated from ultrafiltration flow times sieving coefficient, was low and lower in the citrate group (P = 0.001); it decreased from 10 minutes to 720 minutes of CVVH (P < 0.001) from 16 (2–315) ml/min to 2 (1–13) ml/min for the no anticoagulation group, 12 (5–374) ml/min to 5 (2–11) ml/min for heparin and from 4 (1–25) ml/min to 1 (0–2) ml/min for the citrate group. In the heparin group, there was C5a production across the filter which most decreased over time as compared to other groups.

### Elastase

**Figure 2:**
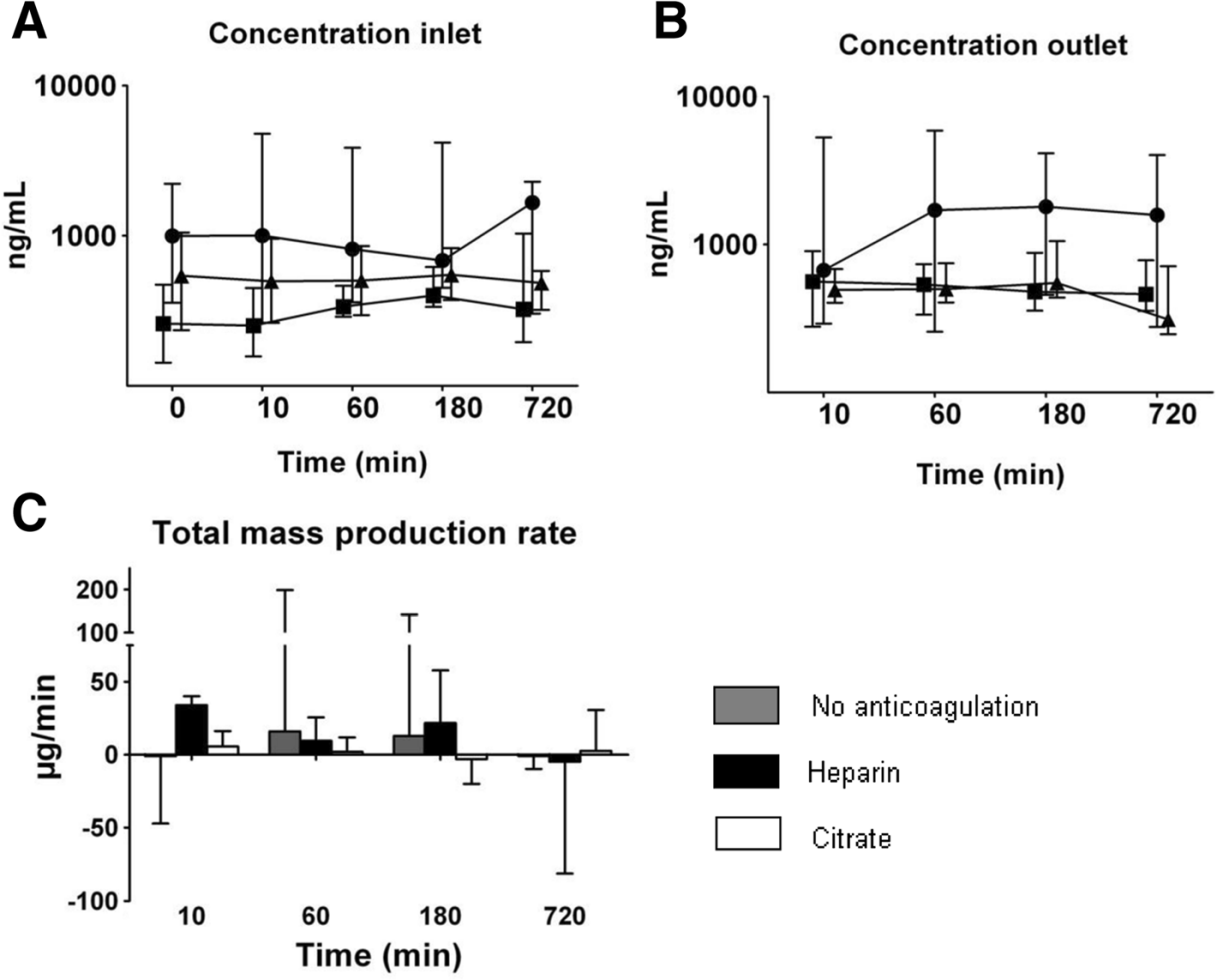
**Concentration of elastase measured at inlet (C**_**i**_**) and outlet (C**_**o**_**) and total mass production rate (Mtp) over time (median and interquartile range).** Results of generalized estimating equations (symbols: ● no anticoagulation ■ heparin ▲ citrate) for elastase in **A.** C_i_: increases over time, most in the no anticoagulation group (P = 0.002 for interaction). **B.** C_o_: increases over time (P = 0.012). **C.** M_tp_: highest in the heparin group (P = 0.049), decreases over time most in the heparin group (P = 0.002 for interaction).

Prior to initiation of CVVH, elastase was detectable in all patients in inlet plasma. Over time, the concentration at inlet increased most in the no anticoagulation as compared to other groups [Figure 2]. The concentration at outlet increased over time, mostly between 10–60 minutes, irrespective of the anticoagulation regimens. The total mass production was higher in the heparin group as compared to the other groups and most decreased in time.

### MPO

**Figure 3:**
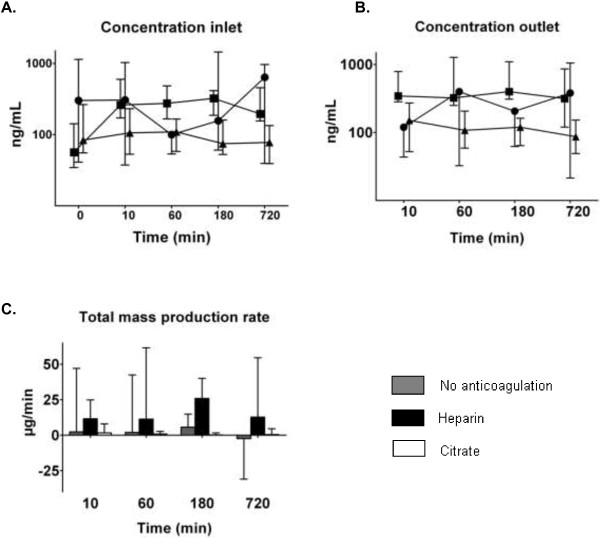
**Concentration of myeloperoxidase (MPO) measured at inlet (C**_**i**_**) and outlet (C**_**o**_**) and total mass production rate (M**_**tp**_**) over time (median and interquartile range).** Results of generalized estimating equations (symbols: ● no anticoagulation ■ heparin ▲ citrate) for myeloperoxidase in **A.** C_i_: increases over time (time, P < 0.001), most in the no anticoagulation group (P < 0.001 for interaction). Note the increase in the heparin group between T = 0 and T = 10 minutes (P = 0.024). **B.** C_o_: highest in the heparin group (P < 0.001) and increases over time (P < 0.001), most in the no anticoagulation group (P = 0.005 for interaction). **C.** M_tp_: highest in the heparin group (P = 0.034).

Prior to initiation of CVVH, MPO was detectable in all patients in inlet plasma. Over time, the concentration at inlet increased over time in all groups, but most in the no anticoagulation group [Figure 3]. There was an increase of MPO between 0 and 10 minutes in the heparin group. The concentration at outlet was highest in the heparin group as compared to the other groups; it increased over time, particularly in the no anticoagulation group. Hence, the total mass production was highest in the heparin group as compared to the others groups.

### Correlations

C5a directly correlated with elastase and MPO concentrations in inlet plasma for all groups and time points together (elastase r_s_ =0.37, P < 0.001; MPO r_s_ =0.30, P < 0.001). No such correlation was found across the filter.

### Sepsis and mortality

For patients with and without sepsis, there were no differences in C5a and elastase concentrations in inlet plasma over time. For MPO however, levels of MPO were higher in the sepsis group (166 (14–3375) ng/ml versus 84 (15–2611) ng/ml in non-sepsis, P = 0.011). Concentrations of C5a did not differ between outcome groups. Plasma concentrations of elastase and MPO, however, were higher in ICU non-survivors than in survivors (elastase: 964 (155–7451) ng/ml versus 394 (28–4225) ng/ml, P < 0.001 and MPO: 174 (29–2670) ng/ml versus 85 (14–1086) ng/ml, P < 0.001).

## Discussion

The present study shows that citrate, in contrast to heparin, does not activate complement and liberate polymorphonuclear neutrophil degranulation products such as elastase and MPO when blood comes into contact with the extracorporeal system and the haemofilter, during CVVH in critically ill patients with AKI. We also suggest that heparin triggers endothelial release of MPO, while CVVH with citrate does not. These findings argue in favour of citrate being superior to heparin as anticoagulation in CVVH in terms of biocompatibility.

At initiation of CVVH and throughout the study, inlet levels of C5a were highest in the citrate group. Levels of C5a at inlet were already higher before the actual administration of citrate-containing substitution fluid (at T = 0 min), suggesting that this observation represents a baseline difference of the citrate compared to the other groups, rather than actual complement activation by citrate in the patient. High inlet levels of C5a could also explain high outlet levels in the citrate group throughout the study. Levels of C5a in the ultrafiltrate were lowest in the citrate group, possible partly due to higher ultrafiltration flows and subsequent dilution, although we cannot fully explain the difference between the citrate groups, both with lower values than in the other groups. However, it is possible that the higher sieving coefficient of C5a observed in the no anticoagulation and heparin group resulted from generation of C5a in the filter and subsequent removal in the ultrafiltrate. The lower sieving coefficient observed when citrate was used, may then argue in favour of less complement activation in the filter and thus improved biocompatibility. Also, the clearance of C5a was low and negligible in the citrate group. This finding is in concordance with another study, where there was hardly any C5a found in the ultrafiltrate and thus virtually no removal by convection of C5a during CVVH particularly when high volumes were used [17]. Possibly, removal of C5a by convection, especially in the citrate group with higher ultrafiltrate rates, was delayed due to incomplete membrane saturation and adsorption of C5a by the filter. Hence, we cannot exclude an effect of higher ultrafiltrate rates on filter and blood contact time. Another factor in the low clearance by convection could be that C5a, even though a small molecule, could have been bound to other substances, thereby exceeding size limits for convective clearance.

Total mass production rate of C5a in the filter was highest and thus most decreased over time in the heparin group. Therefore, increased neutrophilic degranulation evoked by heparin, illustrated by relatively high production of elastase and MPO shortly after connecting the filter, was possibly associated, at least in part, with complement activation in the filter, in contrast to observations during haemodialysis [18,19]. However, there was no correlation between complement activation and neutrophil degranulation across the filter whereas such correlation was present in patient’s plasma. Moreover, there was no evidence for neutrophil degranulation in the filter in the citrate group and there was minimal degranulation in the no anticoagulation group. The latter agrees with the idea that degranulation of neutrophils is, at least partly, calcium dependent and independent of complement activation [10,12,14,18,20]. Our finding that citrate may cause less neutrophil degranulation by heparin during CVVH, is in concordance with studies during intermittent haemodialysis and a single study on CVVH [9,10,12,13,15]. The time course of the release of elastase and MPO seems to be dissimilar in our study, and release may be differently regulated. In other studies, for instance, the use of nafamostat mesylate anticoagulation during haemodialysis completely suppressed release of MPO but did not attenuate increases of elastase [21]. Apart from being stored in granules in neutrophils, MPO is also widely distributed in the endothelium of blood vessels. The observed transient release of endothelium-bound MPO within minutes after bolus administration of heparin to patients agrees with the literature [16) and this phenomenon should be taken into account to consider plasma MPO as an indicator of bioincompatibility [22]. Even though the concentration of MPO in inlet plasma rose early after start of heparin as an indicator of systemic release, the overall concentration did not exceed that in the other groups, thus arguing against substantial endothelial release in our patients.

High serum concentrations of elastase and MPO over time were associated with non-survival, particularly in the no anticoagulation group, as described before in sepsis [23]. An anticoagulation regimen for CVVH that is minimally pro-inflammatory and thus potentially less harmful could be of value for patients. Recently [4], it has been postulated that increased survival with help of citrate as compared to low molecular weight heparin-based CVVH may relate, in part, to better biocompatibility and our findings support this hypothesis. Currently, citrate is often used as an alternative to heparin in patients with a high bleeding tendency. Our results add to the discussion whether citrate should be first choice for regional anticoagulation during CVVH in all critically ill patients with AKI requiring renal replacement therapy.

A limitation of the present study is that the groups were relatively small and not completely formed by randomisation. Furthermore, the citrate group consisted of patients from two different studies, and although patient characteristics between groups did not differ, concentrations of C5a in the ultrafiltrate were lower in patients from the randomized study. This does not invalidate the conclusion that there was less C5a in the ultrafiltrate of patients anticoagulated with citrate compared to the other groups. At baseline there were differences between the anticoagulation groups, by virtue of the study design. The no anticoagulation group consisted of patients without systemic anticoagulation due to a high bleeding tendency and were indeed more severely ill, as demonstrated by higher SAPS II and SOFA scores than in the other groups. These baseline differences, however, may not have affected the proof of principle in these pathophysiological studies.

## Conclusion

In conclusion, citrate confers less filter-induced potentially harmful complement activation and neutrophil degranulation than heparin when used for anticoagulation during continuous venovenous haemofiltration in critically ill patients.

## Competing interests

The authors declare that they have no competing interests.

## Authors’ contributions

LS carried out the measurements of C5a, elastase and MPO, performed the statistical analysis and drafted the manuscript. SN and AG conceived of the study and its design, helped to interpret the data and statistical analysis and drafted the manuscript. NP helped carry out the experiments and RB supervised the conduct of the experiments. BB, AG, PW participated in the design of the study and critically revised the manuscript. All authors read and approved the final manuscript.

## Pre-publication history

The pre-publication history for this paper can be accessed here:

http://www.biomedcentral.com/1471-2369/15/19/prepub

## Supplementary Material

Additional file 1 **The concentrations of C5a, elastase and MPO in patients anticoagulated with citrate from two studies measured at inlet (C**_**i**_**) and outlet (C**_**o**_**); the total mass production rate (M**_**tp**_**) and, for C5a, the concentrations in the ultrafiltrate (C**_**uf**_**) and the sieving coefficient (SC) (median and interquartile range).** Results of generalized estimating equations (symbols: ▲ observational study, n=10 ▼ randomized trial, n=7): A. C5a. C_uf_ was lower in the randomized trial (P<0.001). There were no differences between the citrate groups in C_i_ (P=0.59), C_o_ (P=0.74), SC (P=0.11) or M_tp_ (P=0.48). B. Elastase. There were no differences between the citrate groups in C_i_ (P=0.56), C_o_ (P=0.63) or M_tp_ (P=0.21). C. MPO. There were no differences between the citrate groups in C_i_ (P=0.37), C_o_ (P=0.41) or M_tp_ (P=0.19). Click here for file
