# Supplementary material for: Citrate confers less filter-induced complement activation and neutrophil degranulation than heparin when used for anticoagulation during continuous venovenous haemofiltration in critically ill patients
Source: BMC Nephrol. 2014 Jan 17;15:19. doi: 10.1186/1471-2369-15-19 (PMC3898382; doi:10.1186/1471-2369-15-19)

**Additional file 1.**

**The concentrations of C5a, elastase and MPO in patients anticoagulated with citrate from two studies measured at inlet (C_i_) and outlet (C_o_); the total mass production rate (M_tp_) and, for C5a, the concentrations in the ultrafiltrate (C_uf_) and the sieving coefficient (SC) (median and interquartile range).**

Results of generalized estimating equations (symbols: ▲ observational study, n=10 ▼ randomized trial, n=7): **A.** **C5a**. C_uf_ was lower in the randomized trial (P<0.001). There were no differences between the citrate groups in C_i_ (P=0.59), C_o_ (P=0.74), SC (P=0.11) or M_tp_ (P=0.48). **B.** **Elastase.** There were no differences between the citrate groups in C_i_ (P=0.56), C_o_ (P=0.63) or M_tp_ (P=0.21). **C. MPO.** There were no differences between the citrate groups in C_i_ (P=0.37), C_o_ (P=0.41) or M_tp_ (P=0.19).


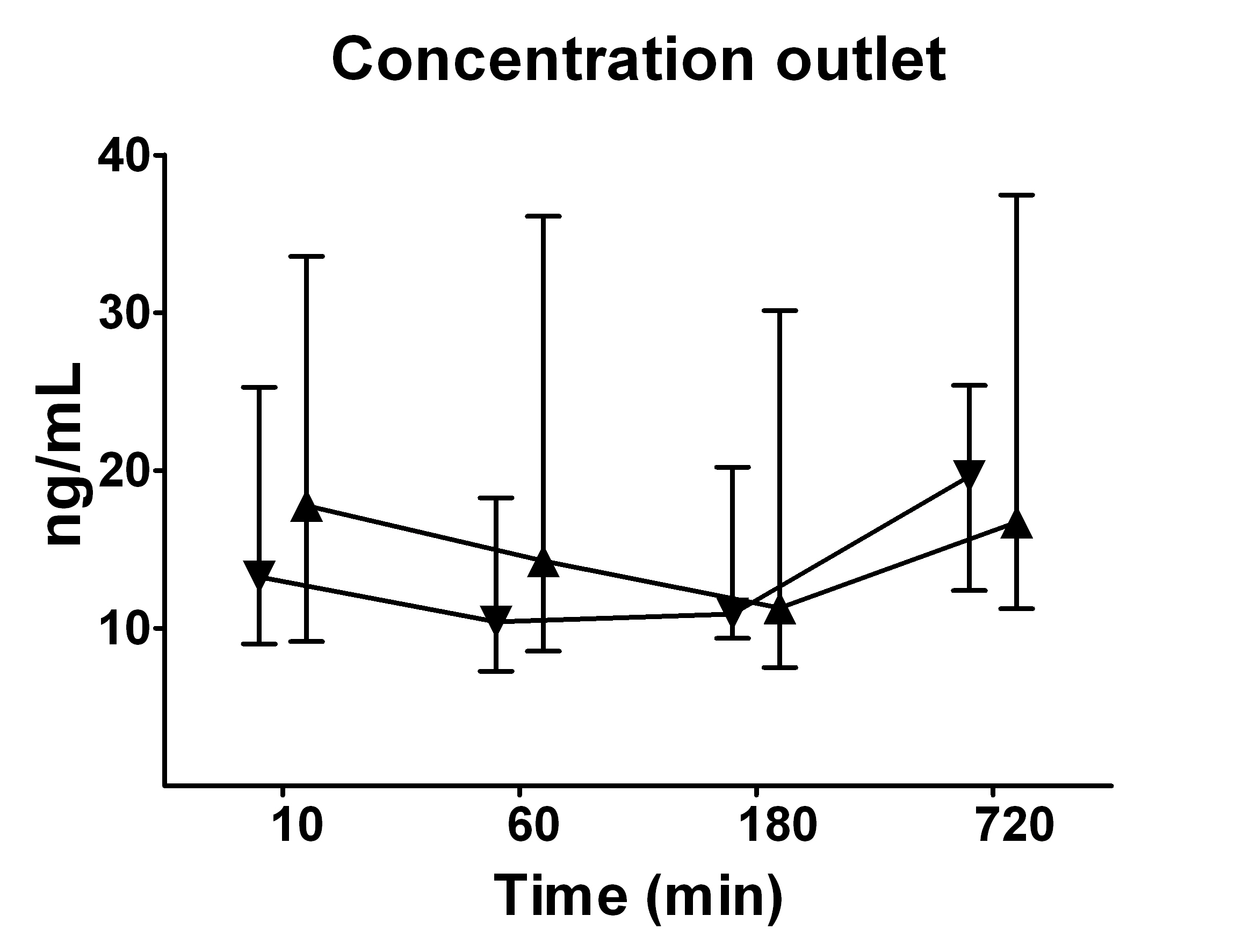
**A. C5a.**

**
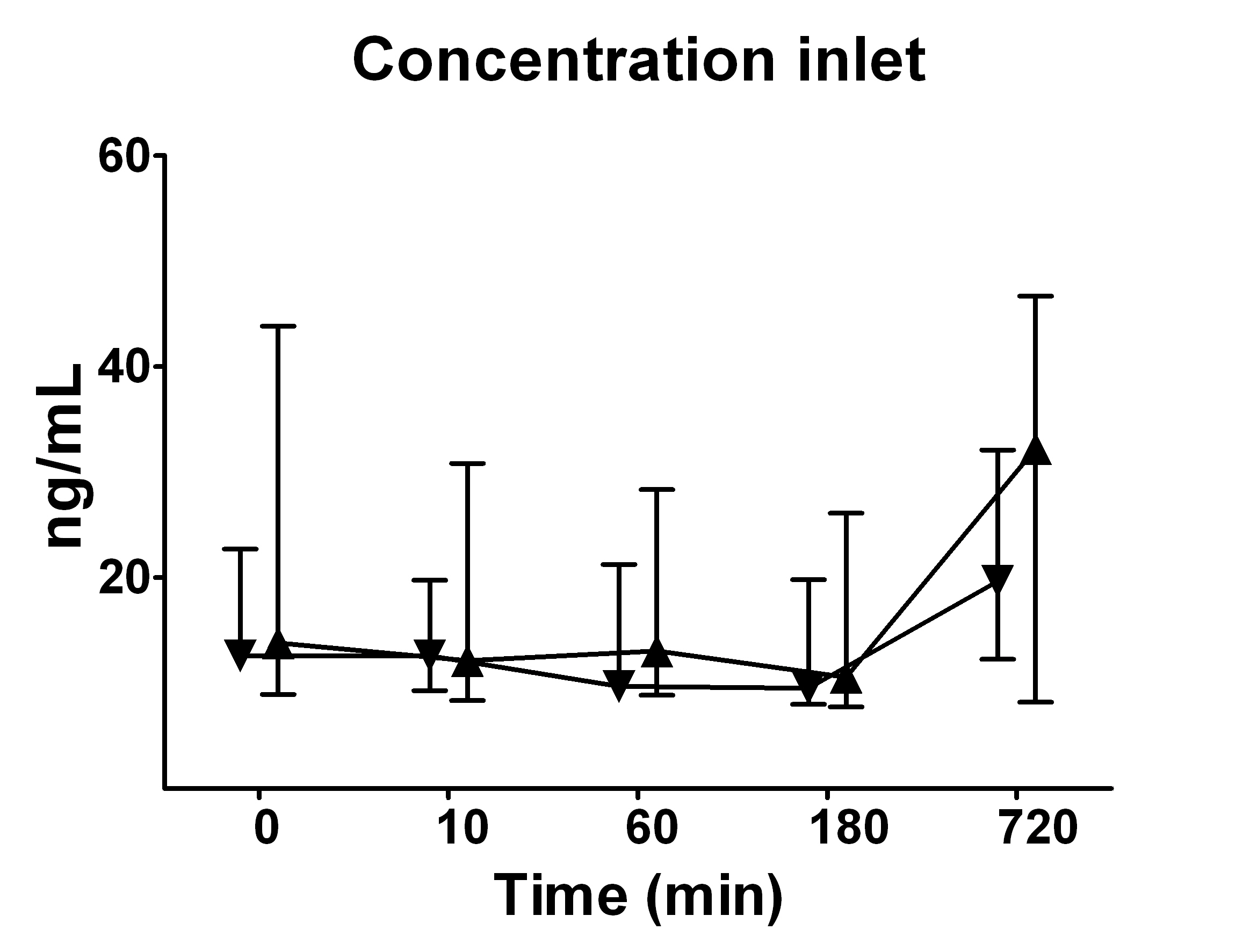
**


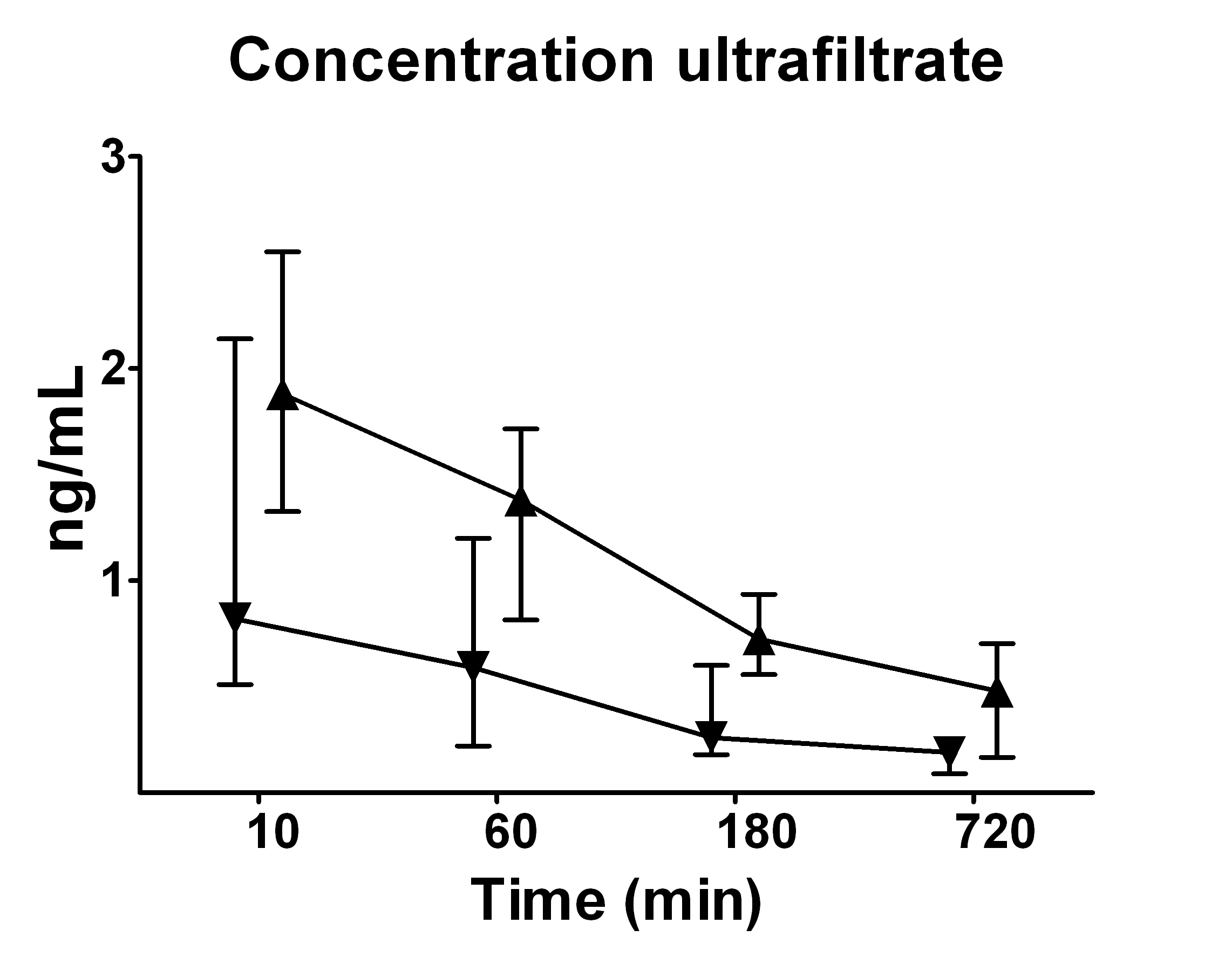

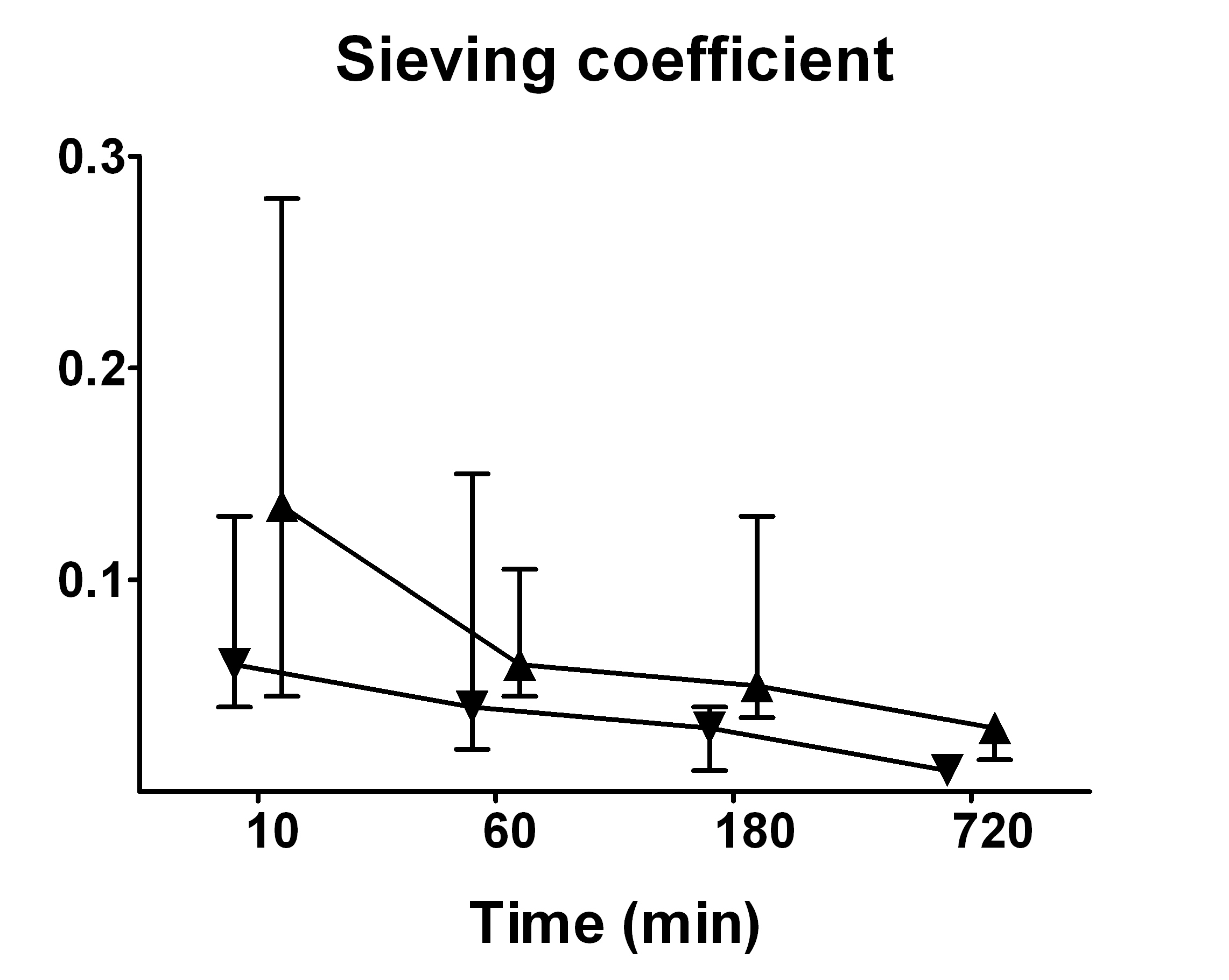


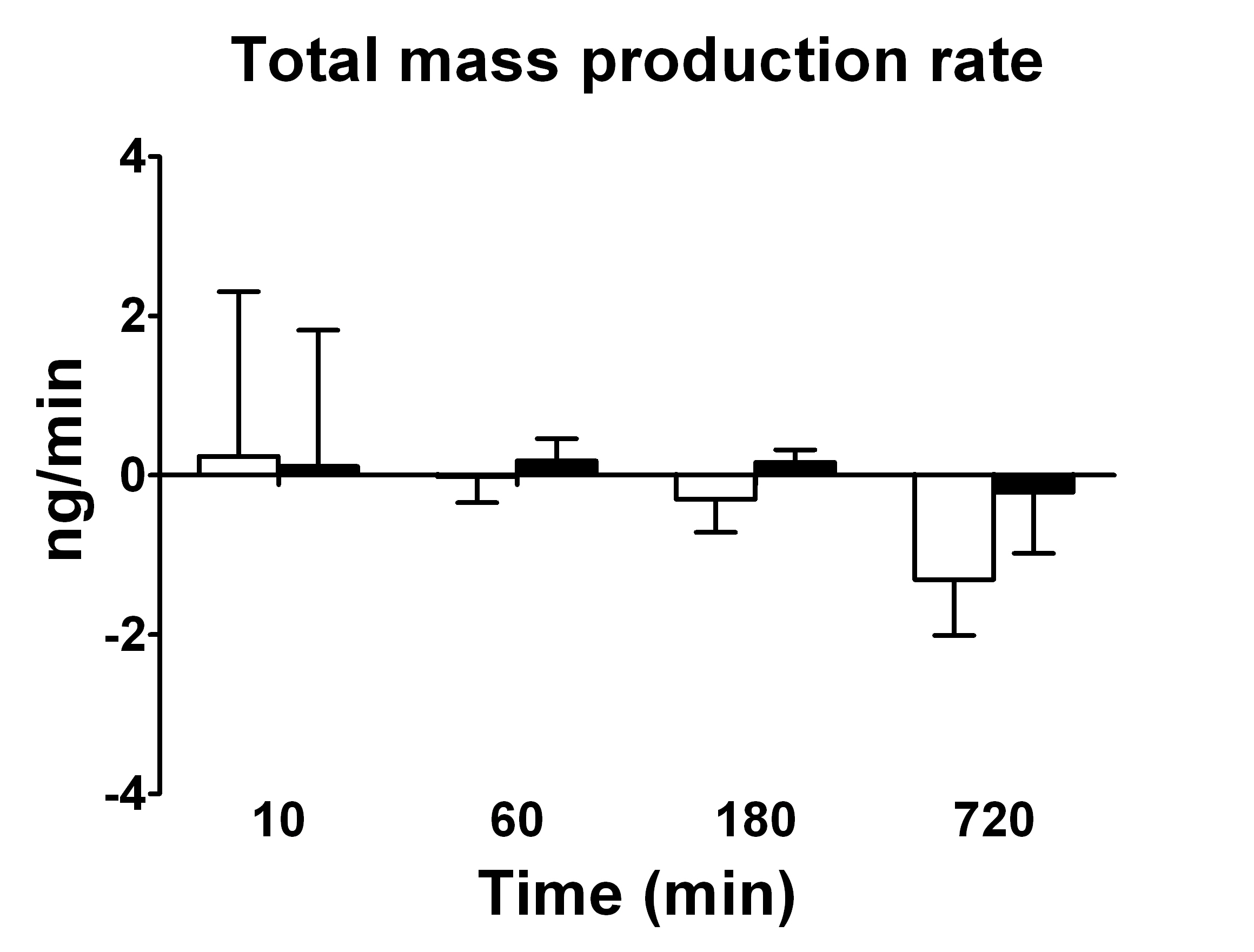


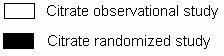


**B. Elastase.**


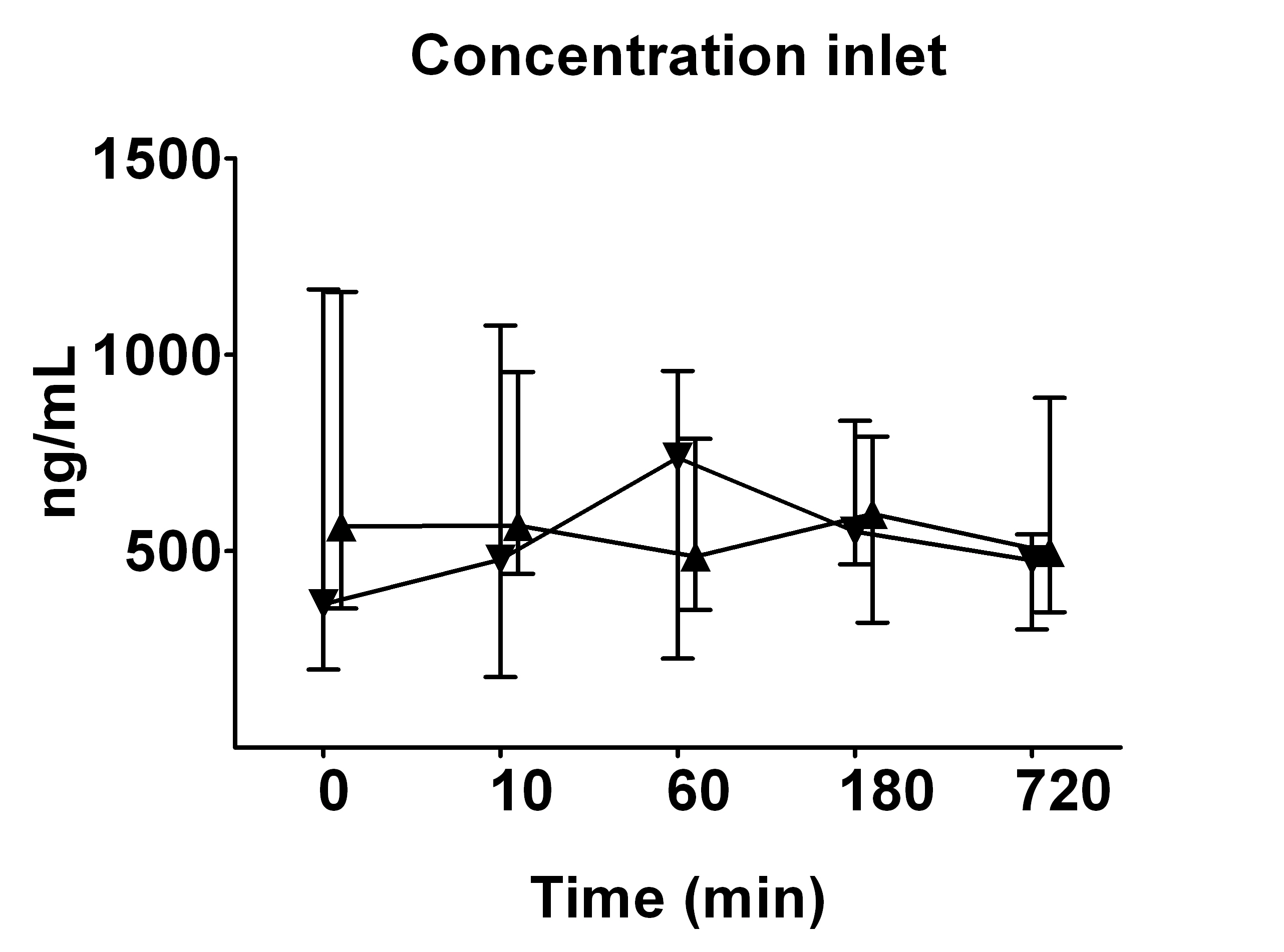

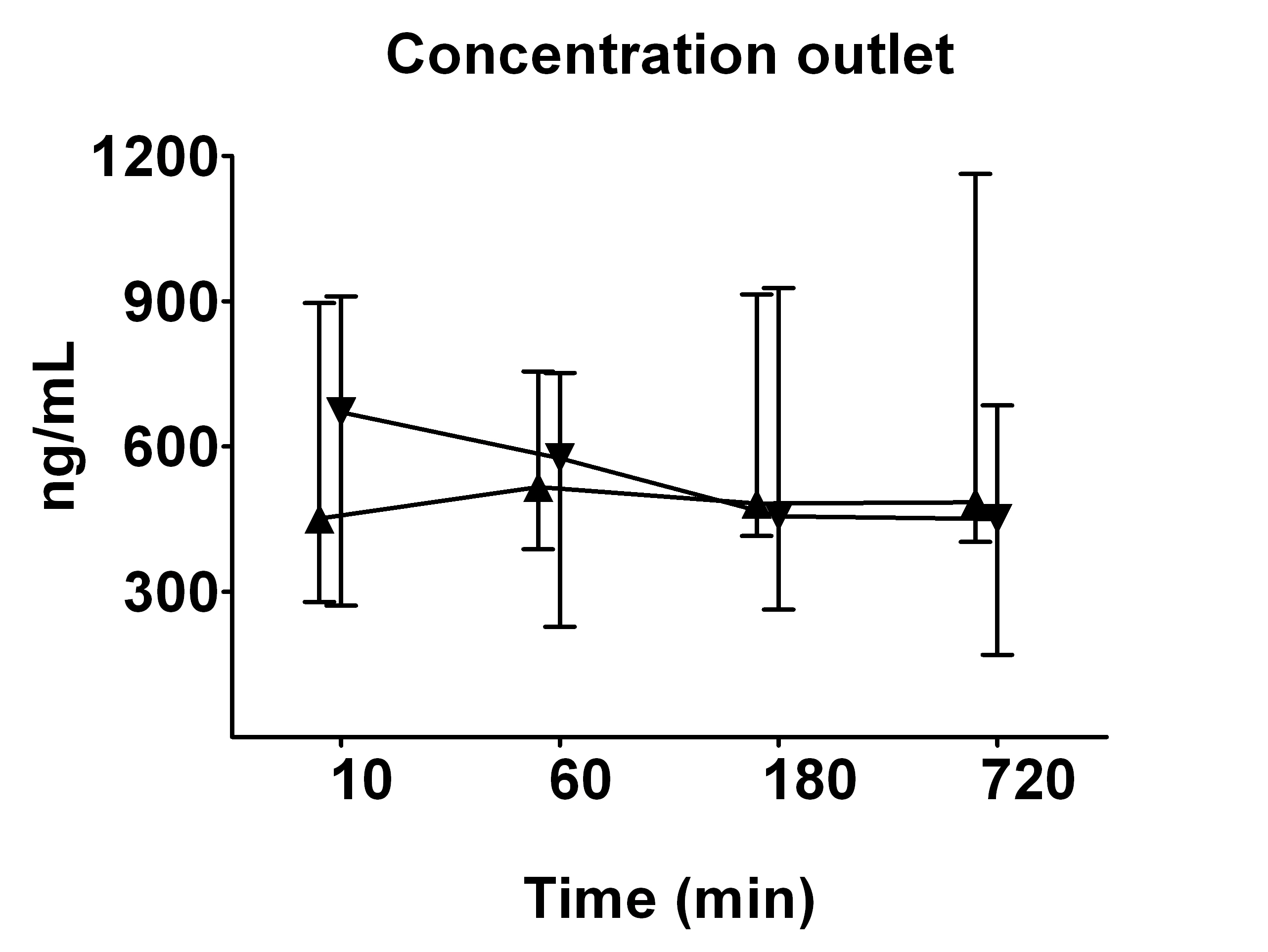


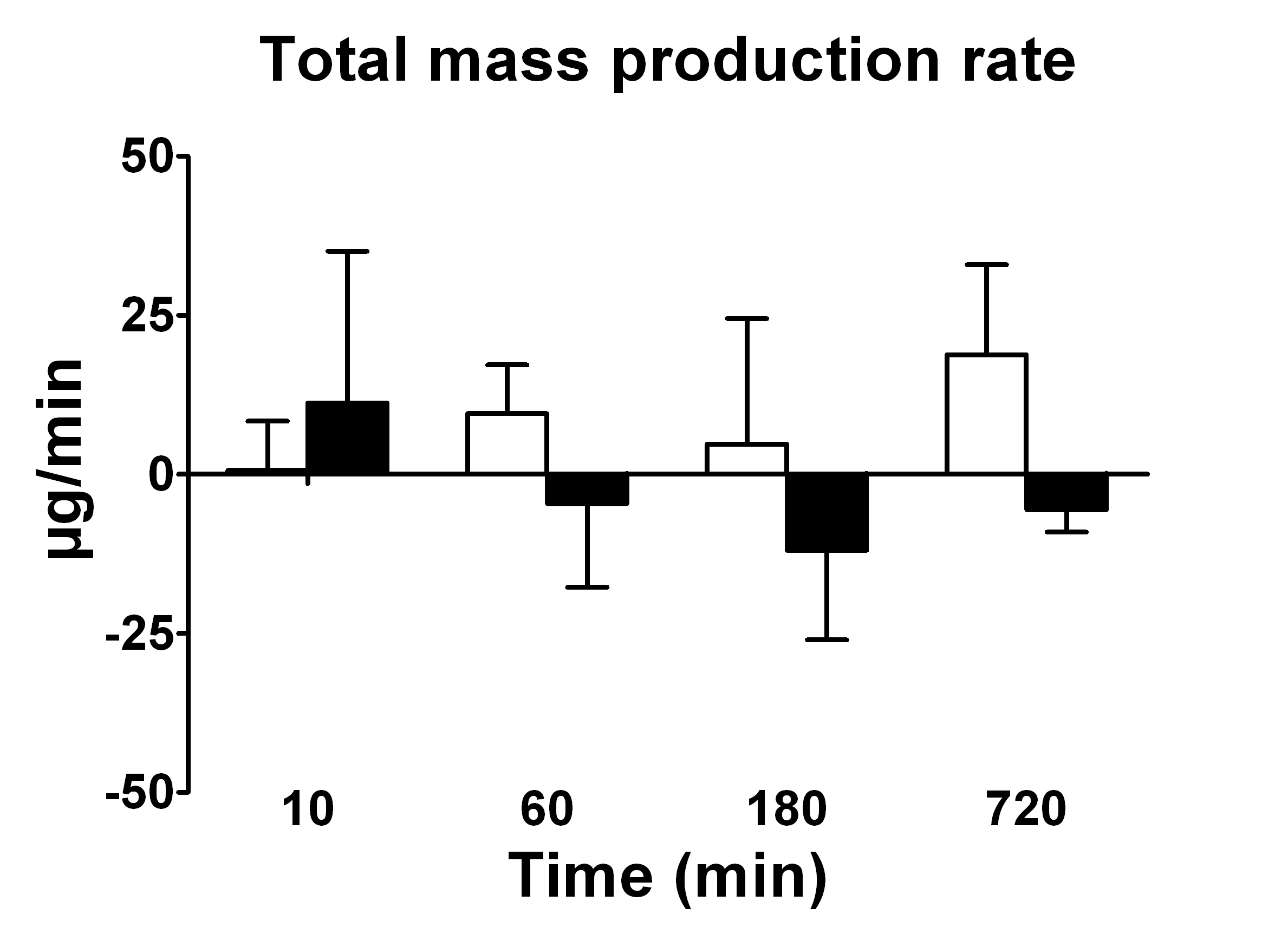


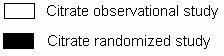


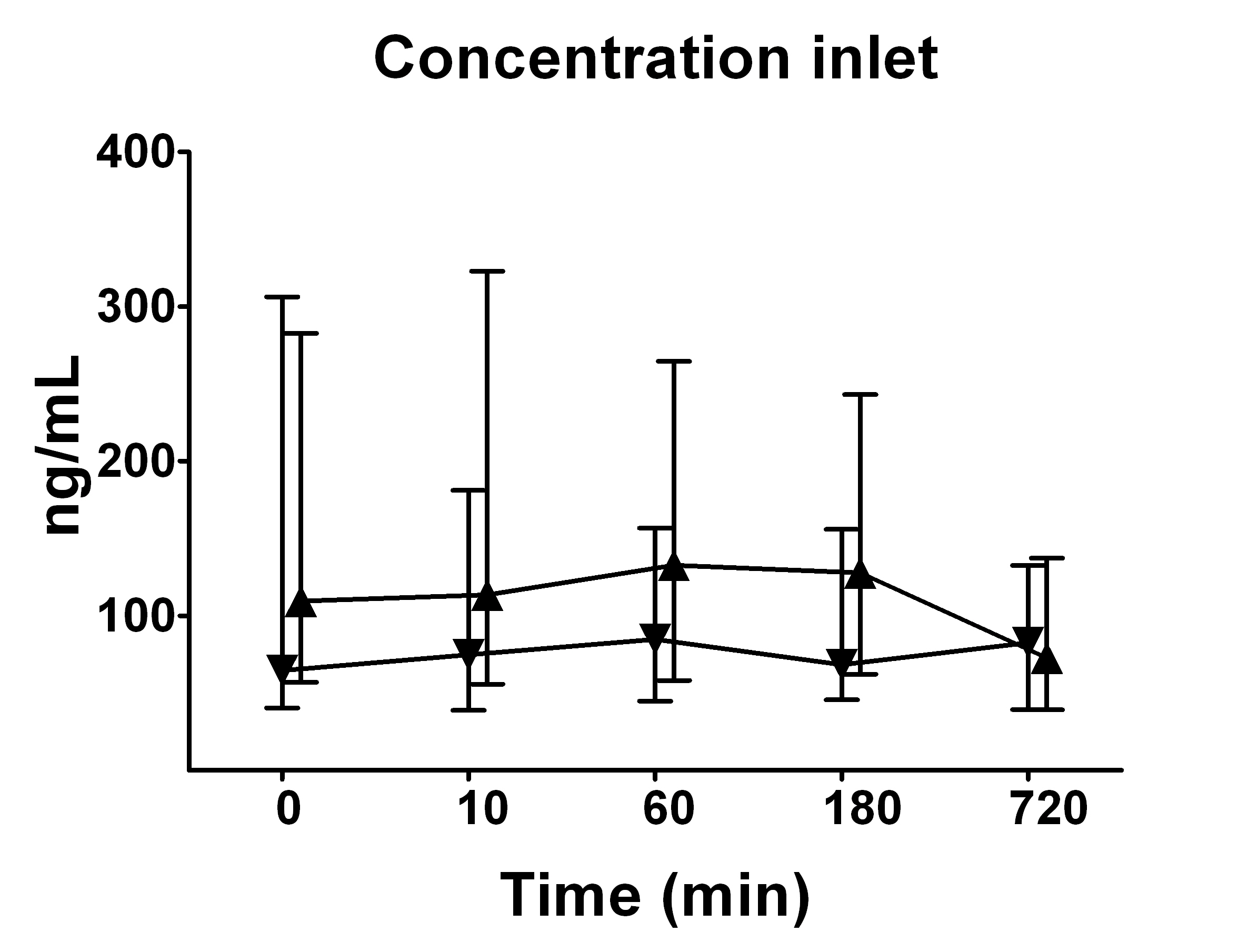

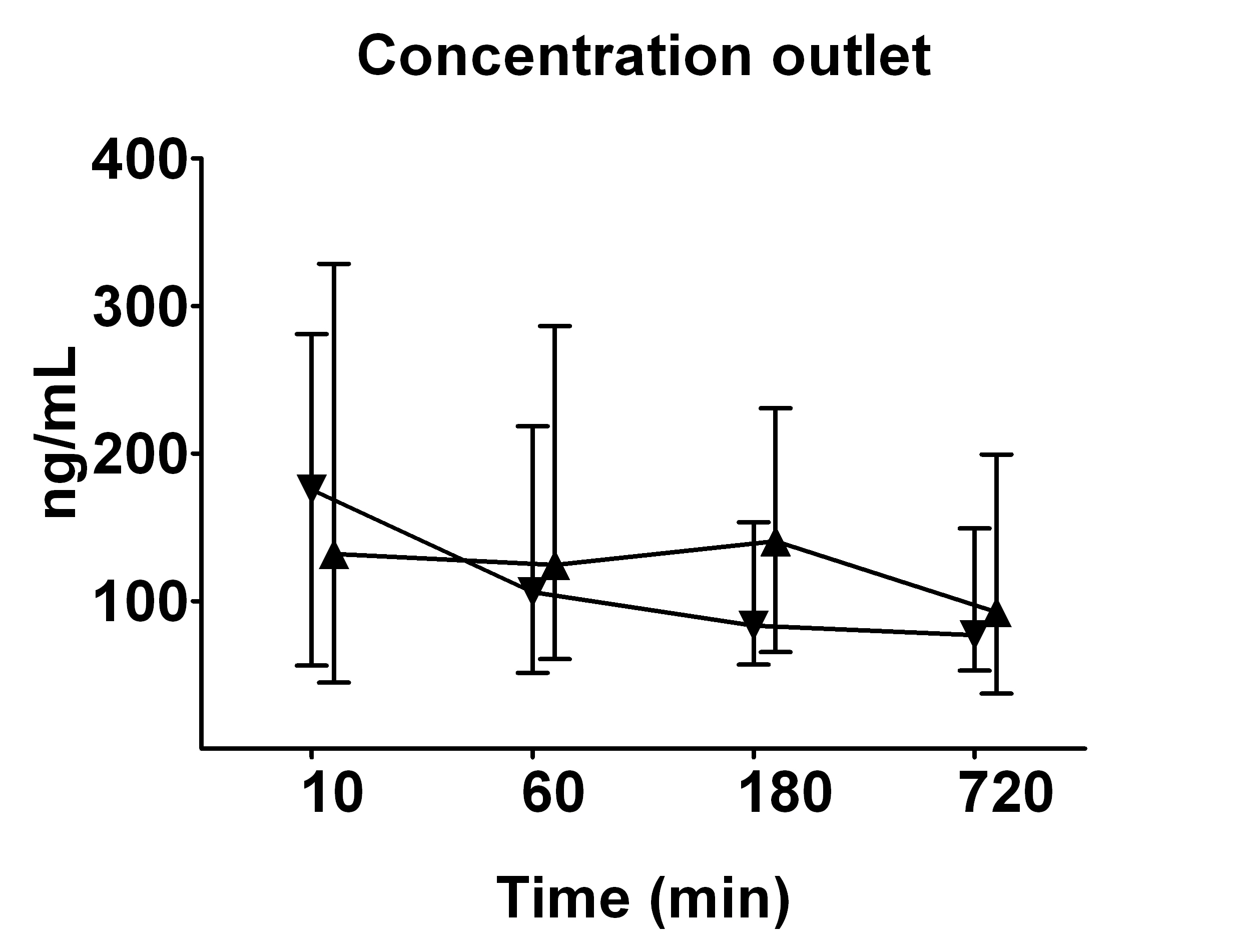
**C. MPO.**


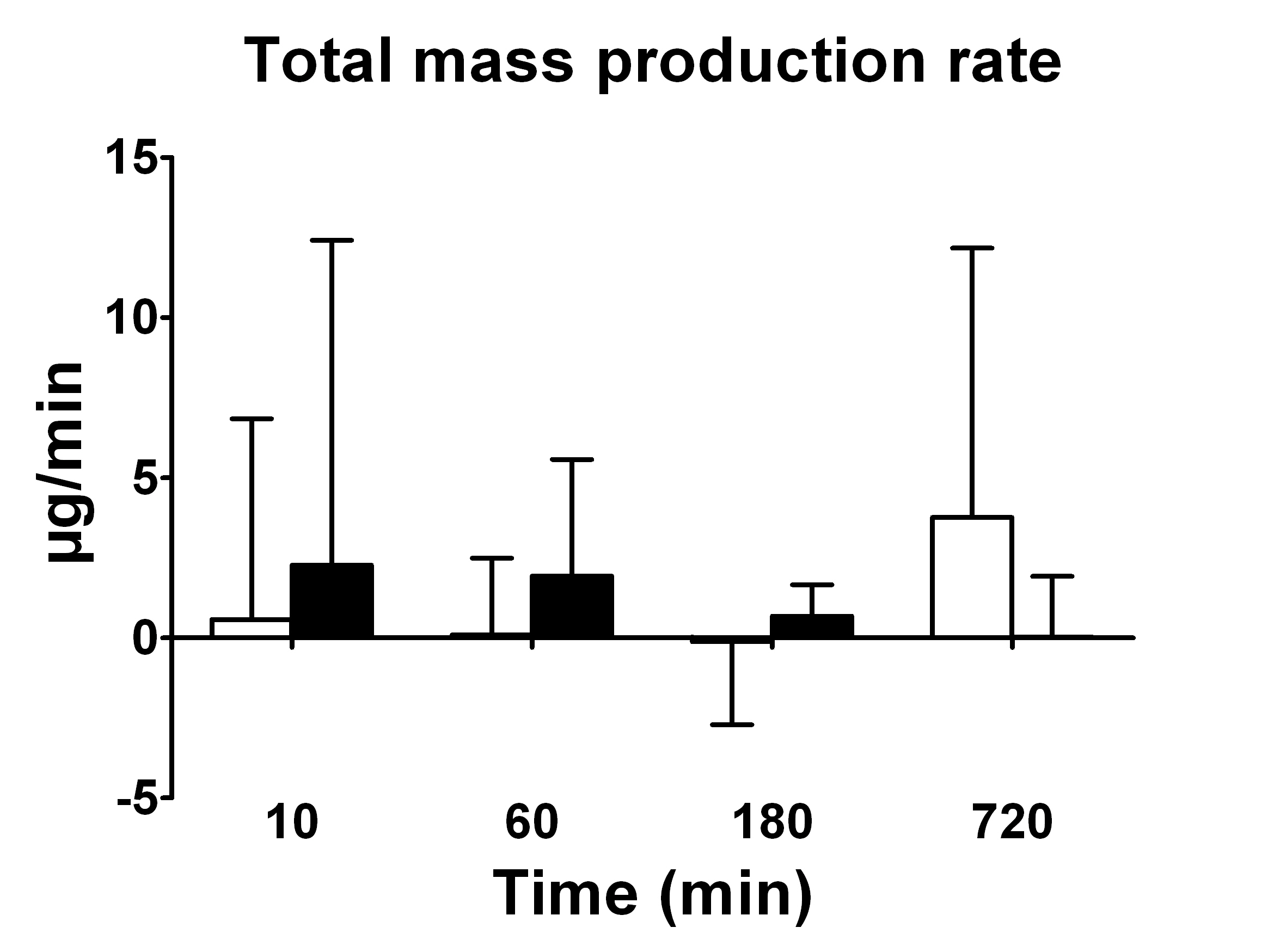


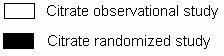

Supplement: Additional file 1 — The concentrations of C5a, elastase and MPO in patients anticoagulated with citrate from two studies measured at inlet (C i ) and outlet (C o ); the total mass production rate (M tp ) and, for C5a, the concentrations in the ultrafiltrate (C uf ) and the sieving coefficient (SC) (median and interquartile range). Results of generalized estimating equations (symbols: ▲ observational study, n=10 ▼ randomized trial, n=7): A. C5a. Cuf was lower in the randomized trial (P<0.001). There were no differences between the citrate groups in Ci (P=0.59), Co (P=0.74), SC (P=0.11) or Mtp (P=0.48). B. Elastase. There were no differences between the citrate groups in Ci (P=0.56), Co (P=0.63) or Mtp (P=0.21). C. MPO. There were no differences between the citrate groups in Ci (P=0.37), Co (P=0.41) or Mtp (P=0.19). [file 1471-2369-15-19-S1.doc]
